# Carrageenans as Sustainable Water-Processable Binders for High-Voltage NMC811 Cathodes

**DOI:** 10.1021/acsaem.3c01662

**Published:** 2023-08-14

**Authors:** Ana Clara Rolandi, Cristina Pozo-Gonzalo, Iratxe de Meatza, Nerea Casado, Maria Forsyth, David Mecerreyes

**Affiliations:** † Institute for Frontier Materials, Deakin University, Melbourne 3125, Australia; ‡ CIDETEC Basque Research and Technology Alliance (BRTA), Paseo Miramon 196, 20014 Donostia-San Sebastian, Spain; § POLYMAT, University of the Basque Country UPV/EHU, Avenida Tolosa 72, Donostia-San Sebastián 20018, Spain; ∥ IKERBASQUE, Basque Foundation for Science, Bilbao 48011, Spain

**Keywords:** biopolymer, carrageenans, NMC811 cathodes, aqueous processing, water-soluble binders, lithium-ion batteries

## Abstract

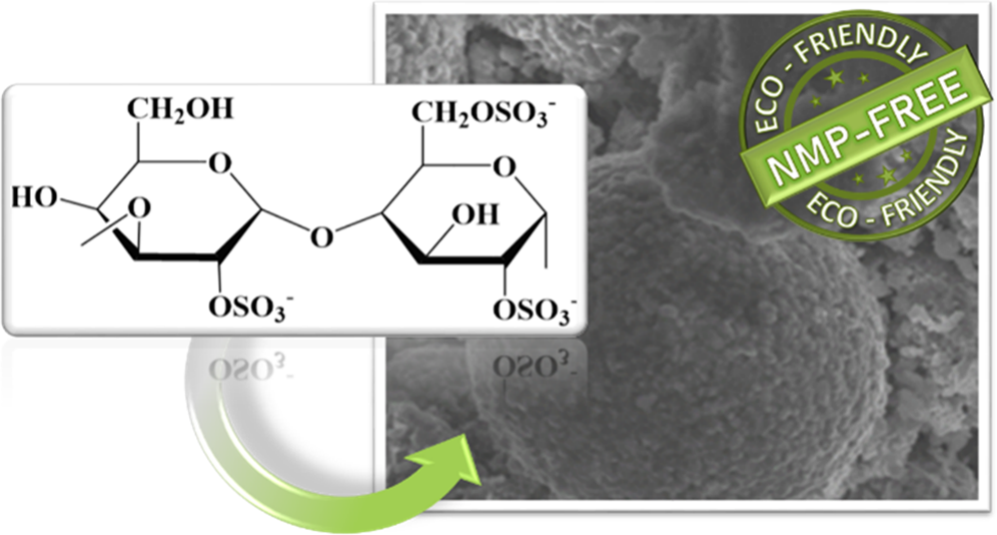

Poly(vinylidene fluoride) (PVDF) is the most common binder for cathode electrodes in lithium-ion batteries. However, PVDF is a fluorinated compound and requires toxic *N*-methyl-2-pyrrolidone (NMP) as a solvent during the slurry preparation, making the electrode fabrication process environmentally unfriendly. In this study, we propose the use of carrageenan biopolymers as a sustainable source of water-processable binders for high-voltage NMC811 cathodes. Three types of carrageenan (*Carr*) biopolymers were investigated, with one, two, or three sulfonate groups (SO_3_^–^), namely, kappa, iota, and lambda carrageenans, respectively. In addition to the nature of carrageenans, this article also reports the optimization of the cathode formulations, which were prepared by using between 5 wt % of the binder to a lower amount of 2 wt %. Processing of the aqueous slurries and the nature of the binder, in terms of the morphology and electrochemical performance of the electrodes, were also investigated. The *Carr* binder with 3SO_3_^–^ groups (3SO_3_^–^*Carr*) exhibited the highest discharge capacities, delivering 133.1 mAh g^–1^ at 3C and 105.0 mAh g^–1^ at 5C, which was similar to the organic-based PVDF electrode (136.1 and 108.7 mAh g^–1^, respectively). Furthermore, 3SO_3_^–^*Carr* reached an outstanding capacity retention of 91% after 90 cycles at 0.5C, which was attributed to a homogeneous NMC811 and a conductive carbon particle dispersion, superior adhesion strength to the current collector (17.3 ± 0.7 N m^–1^ vs 0.3 ± 0.1 N m^–1^ for PVDF), and reduced charge-transfer resistance. Postmortem analysis unveiled good preservation of the NMC811 particles, while the 1SO_3_^–^*Carr* and 2SO_3_^–^*Carr* electrodes showed damaged morphologies.

## Introduction

In order to match the worldwide expansion of electromobility and energy storage from renewable sources, massive research efforts have been undertaken over the past few decades to develop a new generation of lithium-ion batteries (LIBs). The goal is to store more energy and operate for extended periods without degrading or posing safety risks. Despite the binder being only a small component of the battery electrode structure (approx. 5 wt %),^1^ it plays key roles in the battery performance, such as assuring a good distribution of active and conductive materials for optimal lithium diffusion and maintaining the mechanical integrity of the electrode. However, to maximize the battery capacity of the battery, the binder content should be as low as possible (less than 3 wt % of the electrode) while still fulfilling the functions mentioned above.^2^

Unfortunately, the fabrication of cathodes still relies on the use of poly(vinylidene fluoride) (PVDF) as a binder. Alongside the drawback of the fluoropolymer disposal once reaching the end of life of the cell, PVDF has to be dissolved in *N*-methyl-2-pyrrolidone (NMP), which is a toxic and teratogenic solvent,^3^ and requires elevated temperatures for drying; none of those are ideal from an environmental perspective. These facts further increase the cost of battery processing since an expensive recovery system is needed to avoid the release of NMP in the atmosphere.^4^ Therefore, aqueous electrode processing has emerged as an optimistic alternative to reduce the environmental impacts and energy consumption since water is not toxic and evaporates at lower temperatures than NMP.^5,6^

For this reason, many researchers have explored water-based binder systems for the fabrication of cathodes of LIBs,^7−13^ especially biopolymers, where sodium carboxymethyl cellulose (Na-CMC) is the most widely used.^14−16^ Biopolymers are appealing alternatives due to their natural availability, tunability, and relatively lower cost compared to the widely used binder, PVDF. Another interesting biopolymer family is carrageenans, which are water-soluble and linear sulfonated polysaccharides that have been extensively utilized in food and medical applications.^17^ Carrageenans are commercially available as kappa, iota, and lambda carrageenans with one, two, or three sulfonate (3SO_3_^–^) groups, respectively,^18^ being obtained from red seaweeds (*Euchema cottoni*, *Chondrus criptus*, and *Euchema spisosum*). The occurrence of the sulfonate groups in carrageenan is natural, unlike Na-CMC where carboxylic groups are introduced by substitution.^8^ The fact that the sulfonate groups are naturally present in the structure may lead to a more uniform distribution along the chains, potentially providing an enhanced formation of pathways for lithium transport.^19^ Carrageenans have been studied as binders in lithium–sulfur batteries (Li–S),^20^ showing good stability against polysulfide dissolution in the electrolyte, a major issue of Li–S batteries that causes electrode degradation and capacity fading. Sulfonate groups in carrageenan binders are able to capture the polysulfides, avoiding the high-capacity drop and low cycle life observed when PVDF is used as a binder. Furthermore, a recent study applied lambda carrageenan as a binder for silicon anodes,^21^ where the authors reported enhanced adhesion strength, lithium-ion diffusion, and electrochemical performance compared with other common water-soluble binders such as sodium alginate and Na-CMC. The improved behavior was attributed to the number of sulfonate groups in the structure of the biopolymer binder, which effectively accommodated the huge volume changes that silicon suffers during cycling and thus maintained the mechanical integrity of the electrode.

In this article, carrageenans containing a different number of sulfonate groups in their structure are investigated as binders for NMC811 cathodes. At the outset, a typical water-based formulation of 90 wt % of NMC811, 5 wt % of conductive carbon, and 5 wt % of biopolymer binder was explored, comparing its performance with PVDF and Na-CMC, which are processed in NMP and water, respectively. Interestingly, since carrageenans provide high viscosity to the slurry, the formulation could be optimized down to 2 wt % of binder and consequently the proportion of active material in the final electrode increased. As mentioned previously, this is important from an industrial perspective to reduce costs. Finally, the electrochemical performance of the water-based NMC811 cathodes with carrageenan binders was tested using electrodes with a loading of 2.1–2.2 mAh cm^–2^, assessing the impact of the number of sulfonate groups in the structure of the biopolymer.

## Experimental Section

### Materials: Source and Characterization

LiNi_0.8_MnCo_0.1_O_2_ (NMC811, T81RX, Targray), conductive carbon C-NERGY Super C45 (C45, Imerys), and carbon-coated aluminum current collector (CC-Al, Gelon) were used as received. Three carrageenan biopolymers were studied: kappa-carrageenan (1SO_3_^–^*Carr*), iota-carrageenan (2SO_3_^–^*Carr*), and lambda carrageenan (3SO_3_^–^*Carr*) purchased from Sigma-Aldrich. For comparative purposes, poly(vinylidene fluoride) (PVDF, 534 kDa molecular weight, Sigma-Aldrich) was used as a binder with 1-methyl-2-pyrrolidone (NMP, ≥99%, Sigma-Aldrich) as the solvent. As a counterelectrode, graphite anode was prepared for full cell assessment: graphite (HE3, Hitachi) was used as received, and as a binder, a blend of sodium carboxymethyl cellulose (Na-CMC, 250 kDa molecular weight, Sigma-Aldrich) and styrene butadiene rubber (SBR, BM451B, Zeon) was employed. All of the materials were used as received.

The thermal stability of the carrageenan biopolymers was assessed by thermogravimetric analysis (TGA) using a Q500 analyzer (TA instruments) under a nitrogen atmosphere at a rate of 10 °C min^–1^ from 25 to 800 °C. Also, the electrochemical stability of the carrageenans was studied by cyclic voltammetry (CV) in the potential range of 2.0–4.5 V vs Li/Li^+^ at a scan rate of 0.1 mV s^–1^, using a VMP-3 Biologic Instrument. For this, coin cells were assembled using lithium foil as the counter and reference electrodes, which were prepared following the procedure described in the Cathode Electrode: Preparation and Characterization section. The working electrode composition was 50 wt % of carrageenans and 50 wt % of conductive carbon and was free of active material to check for any redox reactions occurring in the binder.

### Cathode Electrode: Preparation and Characterization

Cathode slurries of 50 gr of solids were prepared using water or NMP as the solvent, depending on the binder (carrageenans/Na-CMC or PVDF, respectively). First, the binder was dissolved in the solvent and then the conductive and active material were mixed for 4 h in a mechanical blade mixer at 700 rpm. The final solid-to-liquid ratio was between 1 and 0.9 in all cases (53% solid content).

As mentioned in the Introduction section, apart from exploring different binders, the formulation of the cathode was varied. As outlined in Table 1, the relative amount of binder was decreased, while the active material increased. The proportion of conductive carbon was kept equal for all formulations to avoid further variables under analysis. In the case of the graphite anode, a single composition was employed (94 wt % graphite, 2 wt % conductive carbon, 2 wt % Na-CMC, and 2 wt % SBR latex).

**Table 1:** Different Cathode Formulations Explored in the Present Study

	5 wt % binder	2 wt % binder	1 wt % binder
active material—NMC811 (wt %)	90	93	94
conductive carbon—CB (wt %)	5	5	5
binder (wt %)	5	2	1

Before casting, the final slurries were subjected to rheology tests using a rheometer AR 200ex (TA instruments) in parallel plate geometry, with a 40 mm diameter and a 1 mm gap setting. The dynamic rheological measurements were performed at 25 °C over a shear rate range of 0.1–1000 s^–1^. Then, the slurries were coated on a carbon-coated current collector with a doctor-blade technique at 120 mm min^–1^. The thickness was varied to obtain a loading of 12–13 mg cm^–2^ (2.1–2.2 mAh cm^–2^). After drying the electrodes in a convection oven at 60 °C, they were compacted using a roll-press (DMP solutions) until a porosity of 40% was obtained. The loading of the anodes was balanced to assemble full cells with a negative-to-positive capacity ratio (N/P) of 1.1 (mass loading of anodes 13.2–14.3 mg/cm^–2^).

Peel tests were performed with the calendared electrodes to compare the adhesion strength between different binders and compositions. For this, electrode strips of 2 cm × 9 cm were stuck onto methacrylate plates with a normalized forced and pulled at a 90° angle. The strength value (N m^–1^) is obtained by carrying out the peel test in ambient condition at a crosshead speed of 20 mm min^–1^.

### Coin Cells: Preparation and Electrochemical Characterization

Cathode and anode disks of 16.6 mm and 17.7 mm, respectively, were dried at 120 °C for 16 h under vacuum. The CR2025 cell covers were washed with ethanol in an ultrasonic bath for 15 min and then dried for 1 h at 60 °C. The coin cells were subsequently assembled in a dry room (−40 °C dew point) using the NMC811 cathodes and graphite anodes. As the electrolyte, 100 μL of 1 mol L^–1^ lithium hexafluorophosphate in (1:1 vol %) ethylene carbonate:dimethyl carbonate + 2% vinylene carbonate −99.9% (1 M LiPF_6_ in EC:DMC + 2% VC (1:1)) was used. The separators were glass fiber type (Whatman GF/A) that had been dried at 60 °C for 1 h.

Using a BaSyTec CTS battery test system, galvanostatic charging and discharging cycles were performed on the NMC811|graphite coin cells in the range of 2.8–4.3 V. After 8 h at open circuit potential, a first cycle of formation at 0.1C was carried out and then the electrochemical response was evaluated at various C-rates performing 3 cycles at 0.5, 1, 2, 3, and 5C. Finally, a long-term cycling of 90 cycles at 0.5C was performed. The supplier-provided theoretical capacity of the NMC811 active material (200 mAh g^–1^) was used to determine the C-rate.

With a voltage amplitude of 10 mV and a frequency range of 1 mHz to 1 MHz, electrochemical impedance spectroscopy (EIS) measurements were carried out with a VMP-3 potentiostat (Biologic Science Instrument). The EIS was carried out both after the first formation cycle (pristine) and after the long-term cycling (aged) of the full coin cells (NMC811 cathode and graphite anodes).

Furthermore, the EIS results allow us to derive the Warburg factor (σ) by plotting the real part of the total impedance (*Z*′) against the inverse of square root of the angular velocity (ω^–0.5^), following the Randles eq 1(22)
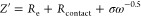
1From the Warburg factor, the lithium-ion diffusion can be found by the Arrhenius eq 2
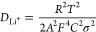
2where *R* is the gas constant (8.314 J K^–1^ mol^–1^), T is the absolute temperature, *A* is the surface area of the electrode, *F* is the Faraday constant (96,500 C mol^–1^), and *C* is the molar concentration of lithium ions. Since the active material is not entirely uniform and the electrode contains voids and pores, both *A* and *C* are complex factors. For this study, constant values of *A* (2.16 cm^2^) and *C* (1 mol cm^–3^) are assumed and the results of *D*_Li_^+^ will be compared qualitatively.

### Microstructural Characterization

The coin cells were disassembled inside a glovebox after the galvanostatic cycling, and the cathodes were then washed with dimethyl carbonate (DMC) to get rid of any residual salts on the surface. Through the use of a field emission scanning electron microscope (FESEM, ULTRA plus ZEISS), the component distribution in both pristine and aged electrodes was observed.

## Results and Discussion

**Figure 1:**
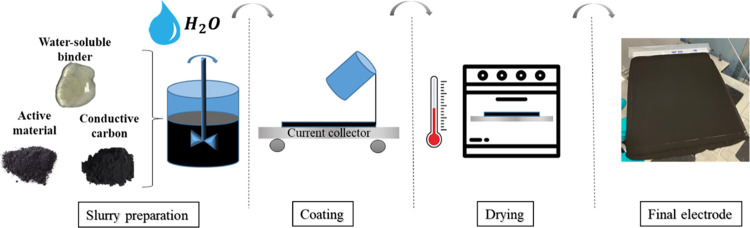
Simplified scheme of the aqueous processing of cathodes for lithium-ion batteries.

In this work, three carrageenan biopolymers with a different number of sulfonate groups in their structure, namely, kappa-carrageenan (1SO_3_^–^*Carr*), iota-carrageenan (2SO_3_^–^*Carr*), and lambda carrageenan (3SO_3_^–^*Carr*), were investigated as aqueous binders for NMC811 cathodes. The first step for the electrode fabrication (Figure 1) is the slurry preparation, where a mechanical mixer is used to form a homogeneous slurry. In comparison to the conventional organic system, aqueous cathode processing is a more environmentally friendly and cost-effective process. During the coating and drying steps, using water as the solvent would substantially reduce the operation costs, compared with the costs associated with the use of NMP as the solvent. This is mainly due to the requirements for a ventilation and recovery system when using NMP, which includes condensation and/or distillation of NMP to avoid its dispersion in the atmosphere. Besides, water is a much cheaper solvent than NMP and has a faster rate of evaporation, reducing the temperature of the drying step and therefore its cost.^23^

**Figure 2:**
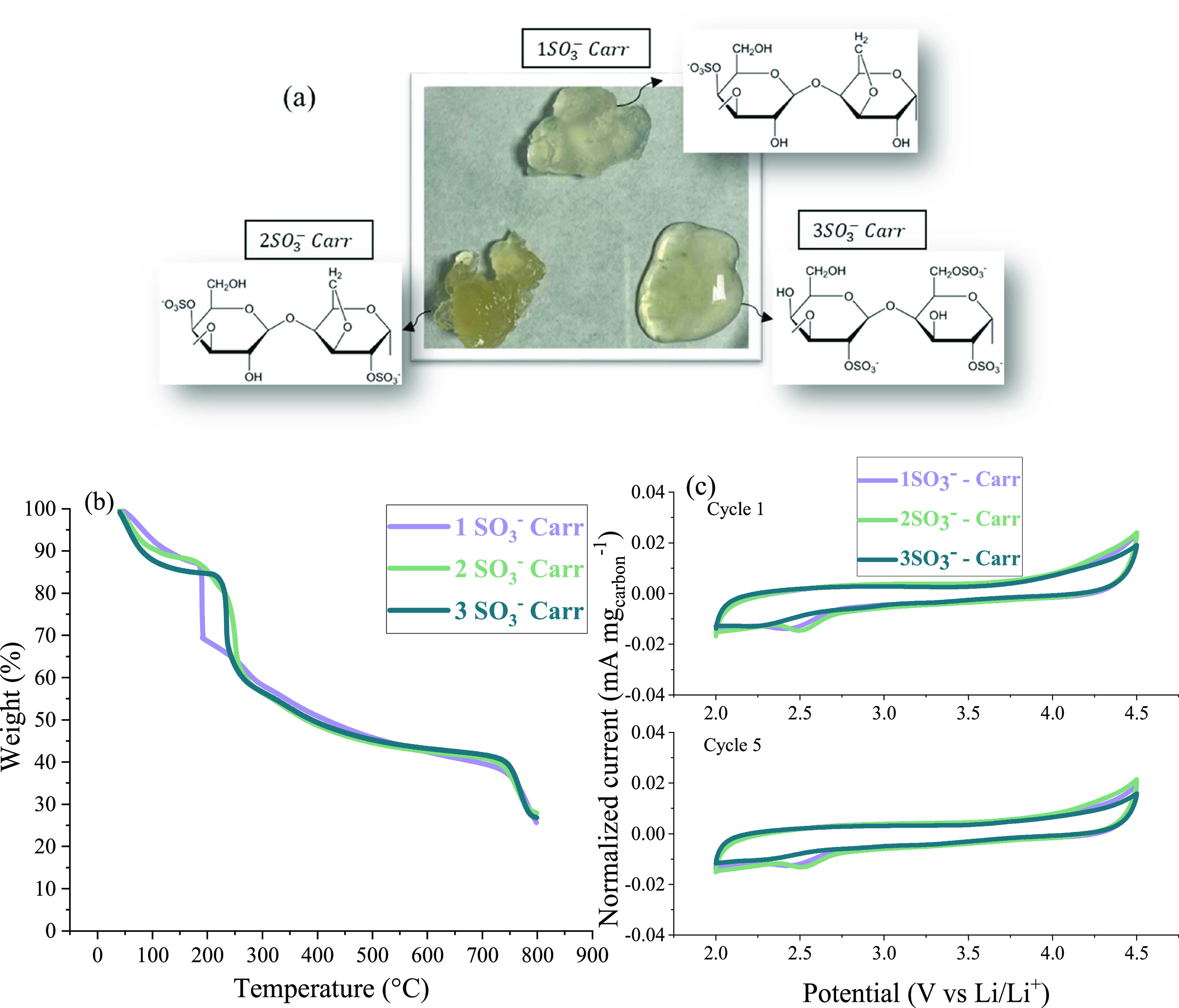
(a) Pictures of the 5 wt % carrageenan in water samples and their chemical structures, showing the different number of sulfonate groups per repeating unit of the 1SO_3_^–^,2SO_3_^–^ and 3SO_3_^–^*Carr*; (b) thermogravimetric analysis (TGA) of the carrageenan biopolymers; and (c) cyclic voltammetry for the potential window of the carrageenan binders. The working electrode is composed of 50 wt % of carrageenans and 50 wt % of conductive carbon, using lithium as the counter and reference electrodes (0.1 mv s^–1^ between 2.0 and 4.5 V vs Li/Li^+^ at room temperature).

As explained in the Experimental Section, the first step of the slurry preparation procedure is to dissolve the binder in the appropriate solvent. Therefore, the three types of carrageenans (1, 2, or 3SO_3_^–^ groups) were dissolved in water until a concentration of 5 wt % was obtained (Figure 2a). The characteristics of the solutions varied, with the solutions prepared with 1SO_3_^–^ and 2SO_3_^–^*Carr* forming a dense mixture, while the one with 3SO_3_^–^*Carr* was less viscous and able to flow. The 3SO_3_^–^*Carr* presents more sulfonate groups per unit and, therefore, larger negative charge. Therefore, to compensate them, it may have a larger interaction with water than the other two and are more prone to form helix structures.

Before testing the carrageenans as binders, two important parameters needed to be assessed, namely, the thermal and electrochemical stability. For this, thermal gravimetric analysis (TGA) was performed, and the corresponding profiles are shown in Figure 2b. The first weight loss of around 10–13 wt % at low temperature (below 100 °C) is attributed to the desorption of water from the polysaccharide structure,^24,25^ and this was more pronounced for the 3SO_3_^–^*Carr* in accordance with its higher water uptake. The onset of the decomposition started at 189 °C for 1SO_3_^–^*Carr* and around 240 °C for the 2SO_3_^–^*Carr* and 3SO_3_^–^*Carr* biopolymers. Therefore, the TGA results proved that all biopolymers are thermally stable at the electrode processing temperature (drying at 120 °C).

Moreover, Figure 2c shows the cyclic voltammetry (CV) data of the cells assembled with a working electrode made of binder and conductive carbon (without NMC811), with lithium foil as the counter and reference electrodes. Five consecutive scans were performed for each binder, between 2.0 and 4.5 V vs Li/Li^+^, to check their electrochemical stability in the voltage range used for cycling. No significant differences were observed between the 1st and 5th cycles, demonstrating the electrochemical stability of the binders upon cycling. However, we noticed that the 1SO_3_^–^*Carr* and 2SO_3_^–^*Carr* biopolymers exhibited a more pronounced oxidation peak at 4.5 V vs Li/Li^+^ than the 3SO_3_^–^*Carr*. Although the normalized current values are small, 3SO_3_^–^*Carr* may be more electrochemically stable than the other two carrageenan biopolymers.

**Figure 3:**
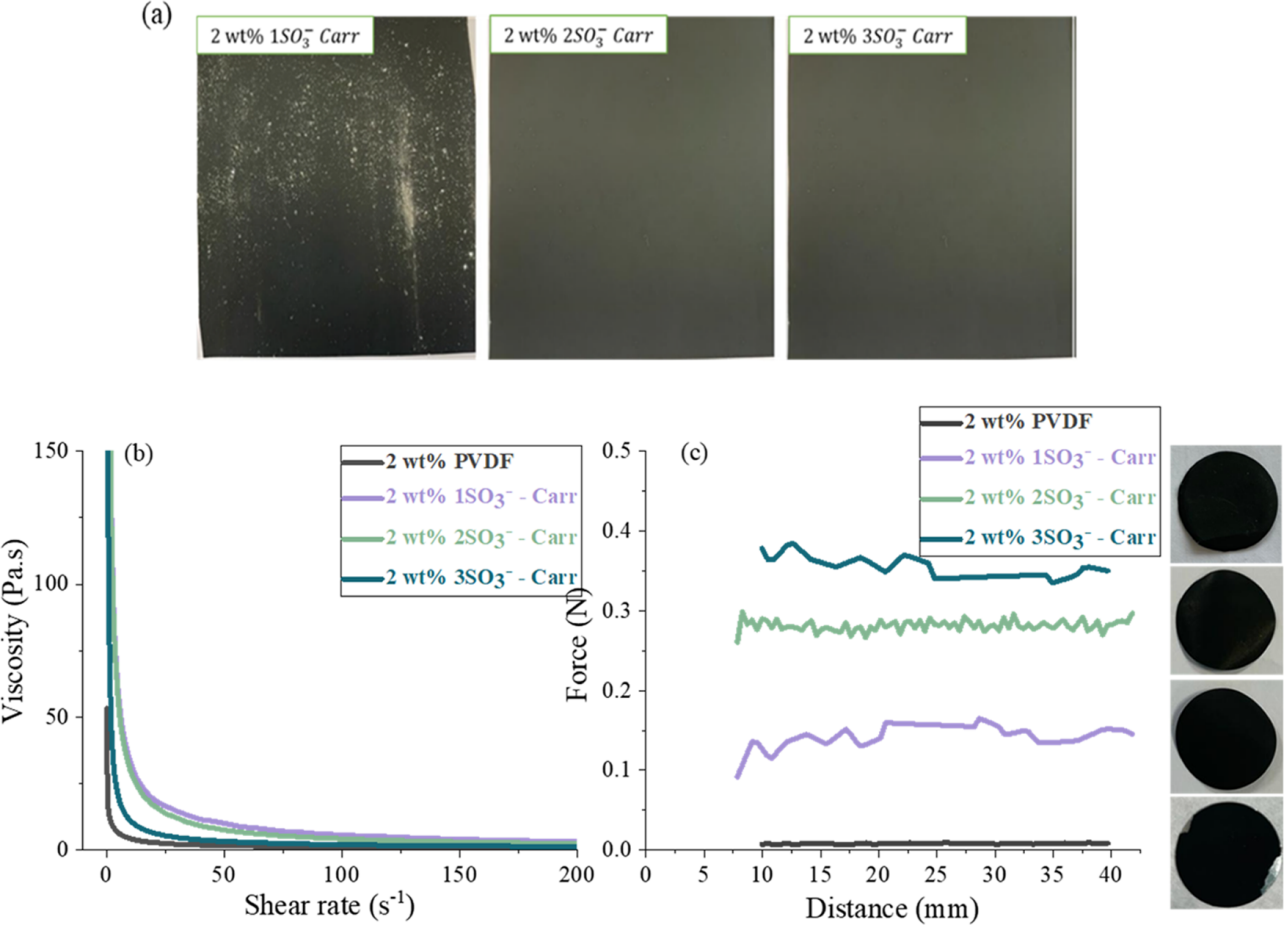
(a) Images of electrodes prepared with the slurries containing the different carrageenan biopolymers with 1, 2, and 3SO_3_^–^ groups per repeating unit; (b) rheology results of the binder slurries vs PVDF, showing the viscosity as a function of shear rate (0.1 and 200 s^–1^) at 25 °C; and (c) peel tests of electrodes coated from the different binder slurries. All samples from the slurries prepared with the 2 wt % binder formulations.

After that, 5 wt % of binder slurries was prepared (Table 1), which is the typical electrode formulation used in lab scale experiments. The final slurries are shown in Figure S1. As expected, the consistency of the binders affected the rheological properties of the electrode slurries. In fact, the 5 wt % 1SO_3_^–^*Carr*- and 2SO_3_^–^*Carr*-based slurries could not be used for coatings since they were too dense to flow over the current collector. Therefore, formulations with 2 wt % of binder were prepared, effectively increasing the active material content to 93 wt % of NMC811. This approach is preferable since maximization of the amount of active material in the formulation is one of the main goals of the battery optimization and manufacturing process. Following the same procedure for the electrode preparation as described above, coatings with each of the 2 wt % of carrageenan binder slurries were successfully coated onto the current collector (Figure 3a), except for the 2 wt % of Na-CMC, which led to a slurry with aggregates and it was discarded for lack of processability. Also, the final electrode coated with the slurry containing 1SO_3_^–^*Carr* appeared inhomogeneous, while the other two (2SO_3_^–^*Carr* and 3SO_3_^–^*Carr*) gave improved coating properties in terms of homogeneity and dispersion of active material and conductive carbon in the slurry. In the case of the 1SO_3_^–^*Carr* binder, only areas showing uniform coating were selected for testing. Following the same procedure, 1 wt % of binder formulation for cathodes was prepared (Table 1). Unfortunately, the slurries with only 1 wt % of polymer binder were unable to disperse the active and conductive material particles, generating agglomerates in the coating (Figure S2). Liu et al.^26^ explained that when the binder content is too low, there is insufficient polymer to form a fully stable layer on the particle surface, causing agglomeration and sedimentation, as observed in the slurries with 1 wt % of binder cathodes. The repercussions will be that lithium conductivity will be hindered and the electrochemical performance diminished. As a conclusion, we established that in the present work, 2 wt % of binder is the optimal formulation since it is the minimum amount that can be applied while still assuring the mechanical and dispersion properties.

Figure 3b shows the rheology results of the 2 wt % binder slurries. As visually observed in Figure 2a, the solutions of 1SO_3_^–^*Carr* and 2SO_3_^–^*Carr* polymers in water at 5 wt % are more dense than the 3SO_3_^–^*Carr* dissolution. This behavior had a clear impact on the rheological properties of the slurries since the 1SO_3_^–^*Carr*- and 2SO_3_^–^*Carr*-based slurries manifested higher viscosities at all shear rates. Furthermore, these two slurries presented more shear-thinning behavior, where the viscosity varies noticeably with the shear rate. In contrast, PVDF and 3SO_3_^–^*Carr* suffered a huge drop in viscosity at low shear rates and then the viscosity was relatively constant as the shear rate increased.

After drying and calendaring the electrodes, peel tests were performed to evaluate the effect of the sulfonate groups on the adhesion strength of the coatings to the current collector. Figure 3c depicts the force (N) as a function of the distance (mm). To calculate the adhesion strength (N m^–1^), an average of the force values is considered. The data indicate that the adhesion strength of all of the coatings containing the 2 wt % binder was greater than the reference PVDF binder electrode (0.3 ± 0.1 N m^–1^) and, therefore, the coating detached from the current collector as observed in Figure 3c. As a comparison, the peel strength of the 5 wt % PVDF binder was measured (8.6 ± 0.9 N m^–1^) and the coating did not detach. Hence, the amount of binder in the 2 wt % PVDF binder electrode was too low to fulfill its function of assuring the mechanical integrity of the electrode. Notwithstanding, the 2 wt % carrageenan binder electrodes exhibit enhanced mechanical properties. The adhesion strength for the 2 wt % 1SO_3_^–^*Carr-*, 2SO_3_^–^*Carr-*, and 3SO_3_^–^*Carr*-based electrodes resulted in 5.7 ± 1.5, 13.6 ± 3.5, and 17.32 ± 1.7 N m^–1^, respectively. The reason why the peel strength of 1SO_3_^–^*Carr* was notably lower compared with the other two *Carr* binders may be due to the inhomogeneities observed during the drying and calendaring steps of the electrode fabrication. On the other hand, 2SO_3_^–^*Carr* and 3SO_3_^–^*Carr* binders yielded improved coatings than achieved with 1SO_3_^–^*Carr*, with better adhesion strengths. Furthermore, the sulfonated groups are expected to establish stronger bonds with the active and conductive particles, enhancing the adhesion between them and with the current collector. Therefore, the improved mechanical strength of the 3SO_3_^–^*Carr* electrode can be attributed to the larger number of free polar functional and sulfonate groups.

**Figure 4:**
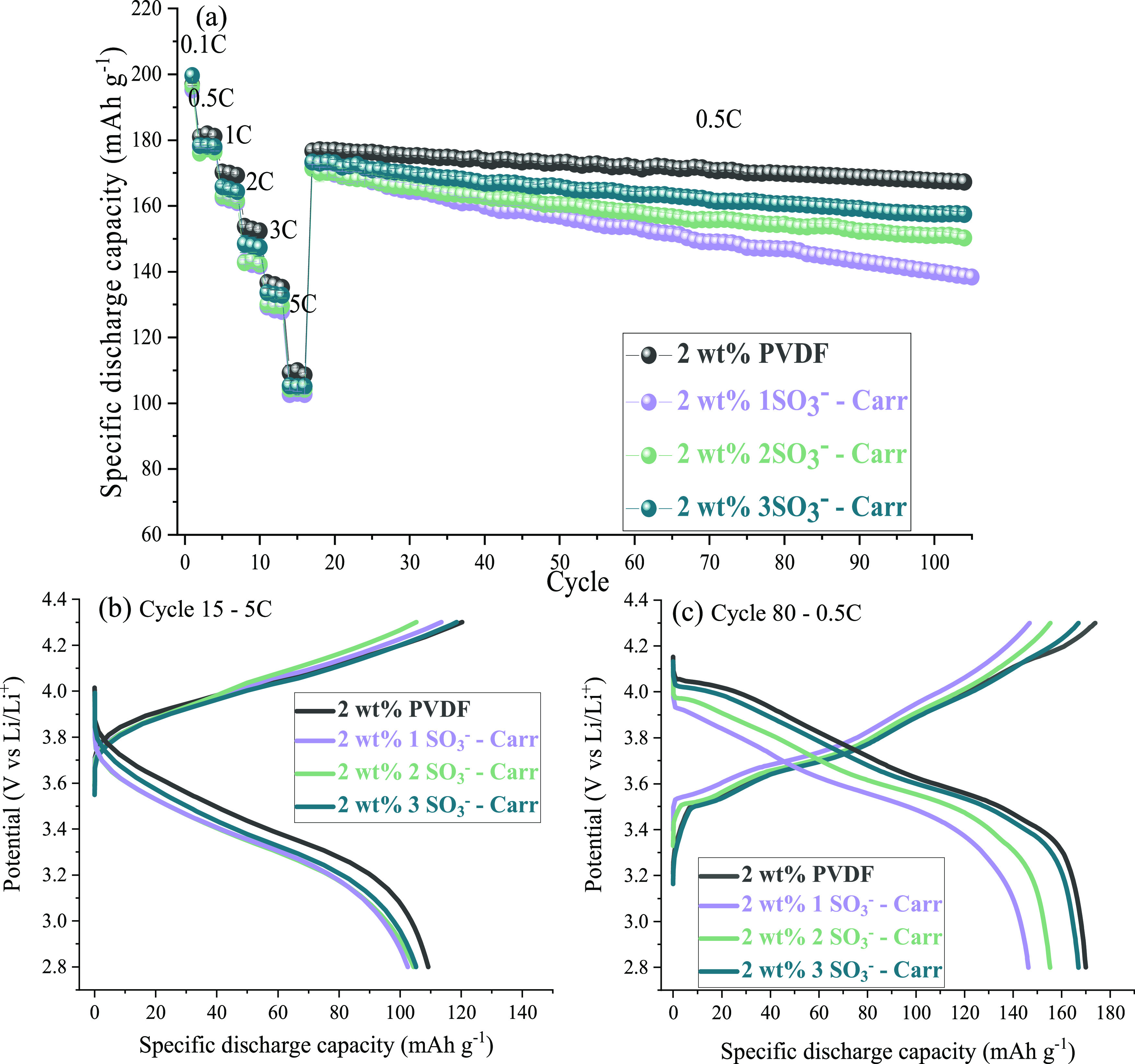
(a) Galvanostatic cycling of full coin cells prepared from the 2 wt % binder cathode formulation (loading 2.1 mAh cm^–2^) using different binders; voltage profiles at (b) 5C, cycle 15 and (C) 0.5C, cycle 80. Potential range: 2.8–4.3 V at 25 °C.

The electrochemical performance of NMC811 cathodes prepared from 2 wt % of binder mixtures was assessed, and the results are shown in Figure 4. In all cases, full coin cells were assembled using graphite anodes and 1 M LiPF_6_ in EC:DMC + 2% VC (1:1) as the electrolyte. To better assess and compare the results, the most relevant data is summarized in Table 2.

**Table 2:** Electrochemical Parameters of the Galvanostatic Cycling Using Electrodes with 2 and 5 wt % of Binder Formulations

	DC^a^ cycle 1 0.1C	DC^a^ cycle 12 3C	DC^a^ cycle 15 5C	DC^a^ cycle 17 0.5C	CR^b^ 90 cycles 0.5C
5 wt % PVDF	200.5	140.4	110.8	188.7	
5 wt % 3SO_3_^–^*Carr*	195.2	120.3	86.0	177.7	
5 wt % Na-CMC	196.1	109.9	65.1	170.1	
2 wt % PVDF	196.9	136.1	108.7	176.7	95
2 wt % 1SO_3_^–^*Carr*	195.3	128.3	102.9	173.1	81
2 wt % 2SO_3_^–^*Carr*	196.4	129.5	104.1	171.4	87
2 wt % 3SO_3_^–^*Carr*	199.6	133.1	105.0	173.4	91

a DC, specific discharge capacity (mAh g^–1^ NMC811).

b CR, capacity retention_90 cycles_ (%) = [DC_Cycle 107_] × [DC_Cycle 17_]^−1^ × 100.

Primarily, during the first cycle of formation at 0.1C, all cells delivered similar discharge capacities (between 195 and 200 mAh g^–1^) with a Coulombic efficiency of 88–89% (Figure S3), including the 2 and 5 wt % binder cathodes. This is attributed to the solid electrolyte formation on the anode side^27^ and therefore seems to be independent of the binder choice for the cathode. However, when increasing the C-rates (Figure 4a), the electrode with 2 wt % of the 3SO_3_^–^*Carr* binder outperformed the other carrageenan binders, being close to the performance of the PVDF-based electrode processed in organic solvent (NMP). The difference between the discharge capacities was not major, as evident from the voltage profiles shown in Figure 4b, although a trend of improved C-rate performance is discerned with the increasing amount of sulfonate groups.

For comparison, Figure S4 depicts the electrochemical performance of several coin cells using the NMC811 cathodes with 5 wt % of different binders: 3*SO*_3_^–^*Carr*, PVDF, and Na-CMC. The results of 5 wt % of 1SO_3_^–^*Carr* and 2SO_3_^–^*Carr* are not shown since, as mentioned before, the slurries could not form a coating. The discharge capacity delivered (Figure S4a) by the PVDF-based cell was higher at all C-rates since it is known that NMC811 is very sensitive toward water.^28^ Nevertheless, the electrodes prepared with 3SO_3_^–^*Carr* as a binder still delivered an adequate performance with only 5% loss of capacity after 30 cycles at 0.5C discharge rate, while the Na-CMC-based cell presented a larger loss of capacity in the same conditions (8%). Considering that the aqueous route is a more environmentally friendly method for the processing of high-energy cathodes, the outcome of the 3SO_3_^–^*Carr* is satisfactory and, moreover, an improvement in comparison to the Na-CMC binder. During the charge–discharge cycling at different C-rates, the 3SO_3_^–^*Carr* binder delivered 120.3 and 86.0 mAh g^–1^ at 3C and 5C, while for PVDF, the discharge capacities were 140.4 and 110.8 mAh g^–1^ at the same C-rates, respectively. This may be due to a higher polarization in the case of the carrageenan binder, which is evident from the voltage profiles at 5C (Figure S4c). These profiles indicate that the 3SO_3_^–^*Carr* binder electrode has higher polarization than the PVDF electrode and could yet be optimized by modification of the cathode formulation. However, 3SO_3_^–^*Carr* once again outperformed the Na-CMC-based cell that only achieved 109.9 and 65.1 mAh g^–1^ at 3C and 5C, respectively.

Therefore, between the different formulations (5 and 2 wt % binders), a notable improvement occurred for the 3SO_3_^–^*Carr* binder. At 3C, the discharge capacity was increased from 120.3 to 133.1 mAh g^–1^ when decreasing the amount of binder from 5 to 2 wt %. Similarly, at 5C, it enlarged from 86.0 to 105 mAh g^–1^. This derived to a capacity increase of 10 and 22% at 3C and 5C, respectively. Therefore, the reduction in the binder proportion not only allowed an enlargement of the proportion of active material in the cathode formulation but also enhanced the electrochemical performance. In contrast, when decreasing the amount of PVDF binder from 5 to 2 wt %, the discharge capacity decreased. Although the loss was minor, an improvement did not occur as it did for the 3SO_3_^–^*Carr* binder. The capacity retention after 90 cycles at 0.5C revealed diverse behavior for the cells using different binders. As evident in Table 2 and Figure 4a, the aqueous electrodes suffered larger capacity decay than the PVDF binder using NMP since NMC811 is highly sensitive when processed with water. However, the capacity retention of the PVDF and 3SO_3_^–^*Carr* binders was not that far apart (95 and 91%, respectively), whereas the other two carrageenans delivered capacity retentions lower than 90%. Voltage profiles of the different electrodes at cycle 80 (0.5C) are depicted in Figure 4c. The 3SO_3_^–^*Carr* and 2SO_3_^–^*Carr* binders delivered less discharge capacity than the 3SO_3_^–^*Carr* and PVDF cathode cells at 0.5C, as a consequence of the increased polarization resistance during the discharge step. The improved performance of the 3SO_3_^–^*Carr* binder-based cell can be attributed to the higher peel strength and better dispersion properties. Furthermore, the sulfonate groups can be acting as lithium carriers during the charge and discharge of the battery. Therefore, by having a higher number of sulfonate groups in the structure, the lithium mobility may be boosted through ionic pathways.^29^ In addition, the larger number of sulfonate groups present in the 3SO_3_^–^*Carr* binder may contribute to the protection of the NMC811 particles against water, achieving a more stable cycling than the 1SO_3_^–^*Carr* and 2SO_3_^–^*Carr*. This effect could be similar to the one observed by Heidbüchel et al.^30^ that have recently reported the positive impact of the addition of Li_2_SO_4_ during the aqueous processing of NMC811 cathodes since a protective coating around the active material was observed by XPS measurements.

**Figure 5:**
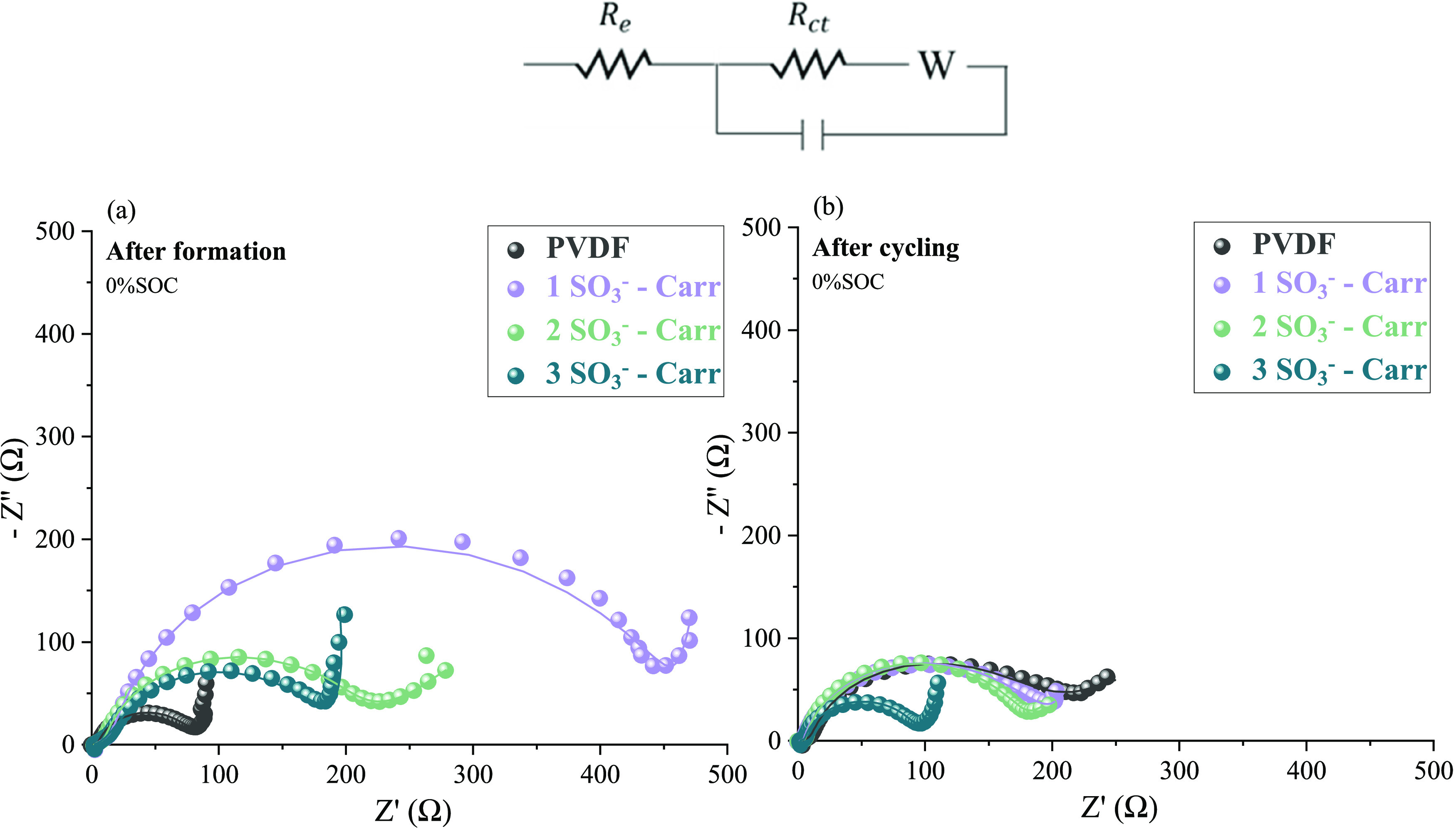
Nyquist plots resulting from the EIS measurements on full coin cells (NMC811|graphite) (a) after the formation step and (b) at the end of cycling (C-rate cycling and 90 cycles at 0.5C).

EIS measurements were conducted on the full coin cells (2 wt % binder formulation NMC811 cathodes vs graphite anodes) after the formation cycle and after cycling (C-rate and 90 cycles at 0.5C). The corresponding Nyquist plots are presented in Figure 5a,b, respectively, fitted with the equivalent circuit as shown on top of the figure. To better understand the variability in the electrochemical behavior, the most relevant data is presented in Table S1. The electrolyte resistance (*R*_e_) is represented by the intersection of the curve with the *Z*′ axis, which is alike for all Nyquist curves (around 1–3 Ω). The following semicircle is assigned to the double layer process of the charge-transfer resistance (*R*_ct_), where significant differences can be observed. After the formation cycle, the *R*_ct_ shows the lowest value for the PVDF cell (79.7 ± 4.2 Ω) and then the *R*_ct_ decreased when increasing the number of sulfonate groups: 435.8 ± 5.6, 205.9 ± 13.5, and 190.3 ± 8.1 Ω for the 1SO_3_^–^*Carr*-, 2SO_3_^–^*Carr*-, and 3SO_3_^–^*Carr*-based cells, respectively. Therefore, the 3SO_3_^–^*Carr* binder presented the lowest value of all of the carrageenan binders, which is in agreement with the galvanostatic cycling results. This is likely a consequence of the higher number of sulfonate groups, such that the lithium-ion conductivity was boosted and the *R*_ct_ reduced. Surprisingly, the *R*_ct_ decreased for the carrageenan cells following the cycling (i.e., the semicircle reduced its diameter), showing values of 171.2 ± 20.2, 169.5 ± 15.7, and 93.1 ± 4.6 Ω for the 1SO_3_^–^*Carr-*, 2SO_3_^–^*Carr*-, and 3SO_3_^–^*Carr*-based cells, respectively. However, the *R*_ct_ of the PVDF cell increased significantly over cycling. Tang et al.^31^ also noticed a reduction of the *R*_ct_ process when using chitosan oligosaccharides for Li_2_ZnTi_3_O_8_ electrodes. The reduction of the impedance was attributed to the formation of more charge-transfer sites during cycling and therefore the improvement in the diffusion parameters. Finally, at high frequencies, the diffusion processes take place, described by the spike line that follows the semicircle; a steeper slope of the curve means that the diffusion of lithium ions is more effective. Following both the formation step and the cycling stages, the 3SO_3_^–^*Carr* yielded the largest slope.

To explore more in detail the diffusion processes that take place at low frequencies, the EIS data was fitted using the Randles equation. The real part of the total impedance (*Z*′) was represented as a function of the angular velocity (ω^–0.5^) (Figure S5), where the slope represents the Warburg factor (σ) and the values are exhibited in Table S1. With the Arrhenius equation, the coefficient of lithium ions (*D*_Li_^+^) can be obtained. The *D*_Li_^+^ for the after-formation steps (Figure S5a) resulted in 3.0 × 10^–13^, 1.4 × 10^–14^, 8.0 × 10^–15^, and 8.5 × 10^–14^ cm^2^ s^–1^ for PVDF, 1SO_3_^–^*Carr*, 2SO_3_^–^*Carr*, and 3SO_3_^–^*Carr*, respectively. The PVDF electrode showed the highest diffusion of lithium ions, followed by the 3SO_3_^–^*Carr* cell, which is in agreement with the galvanostatic cycling results. The difference between the PVDF and the carrageenan cells is probably due to the degradation of the NMC811 active material when in contact with water. However, after the cycling (Figure S5b), the cells showed the following *D*_Li_^+^ values: 1.2 × 10^–14^, 1.4 × 10^–14^, 1.7 × 10^–14^, and 2.3 × 10^–13^ for PVDF, 1SO_3_^–^*Carr*, 2SO_3_^–^*Carr*, and 3SO_3_^–^*Carr*, respectively. While the organic PVDF, 1SO_3_^–^*Carr*, and 2SO_3_^–^*Carr* exhibited similar values of lithium diffusion, the 3SO_3_^–^*Carr* revealed a *D*_Li_^+^ one order of magnitude higher. This striking outcome evidenced the enhanced lithium diffusion conferred by the 3SO_3_^–^*Carr* binder, attributed to the larger amount of sulfonate groups in the electrode that can act as lithium carriers, boosting the conductivity. In conclusion, the reduced *R*_ct_ and enhanced lithium diffusion of the 3SO_3_^–^*Carr* binder led to the improvement in electrochemical performance and this may also be related to the improved mechanical and rheological properties.

**Figure 6:**
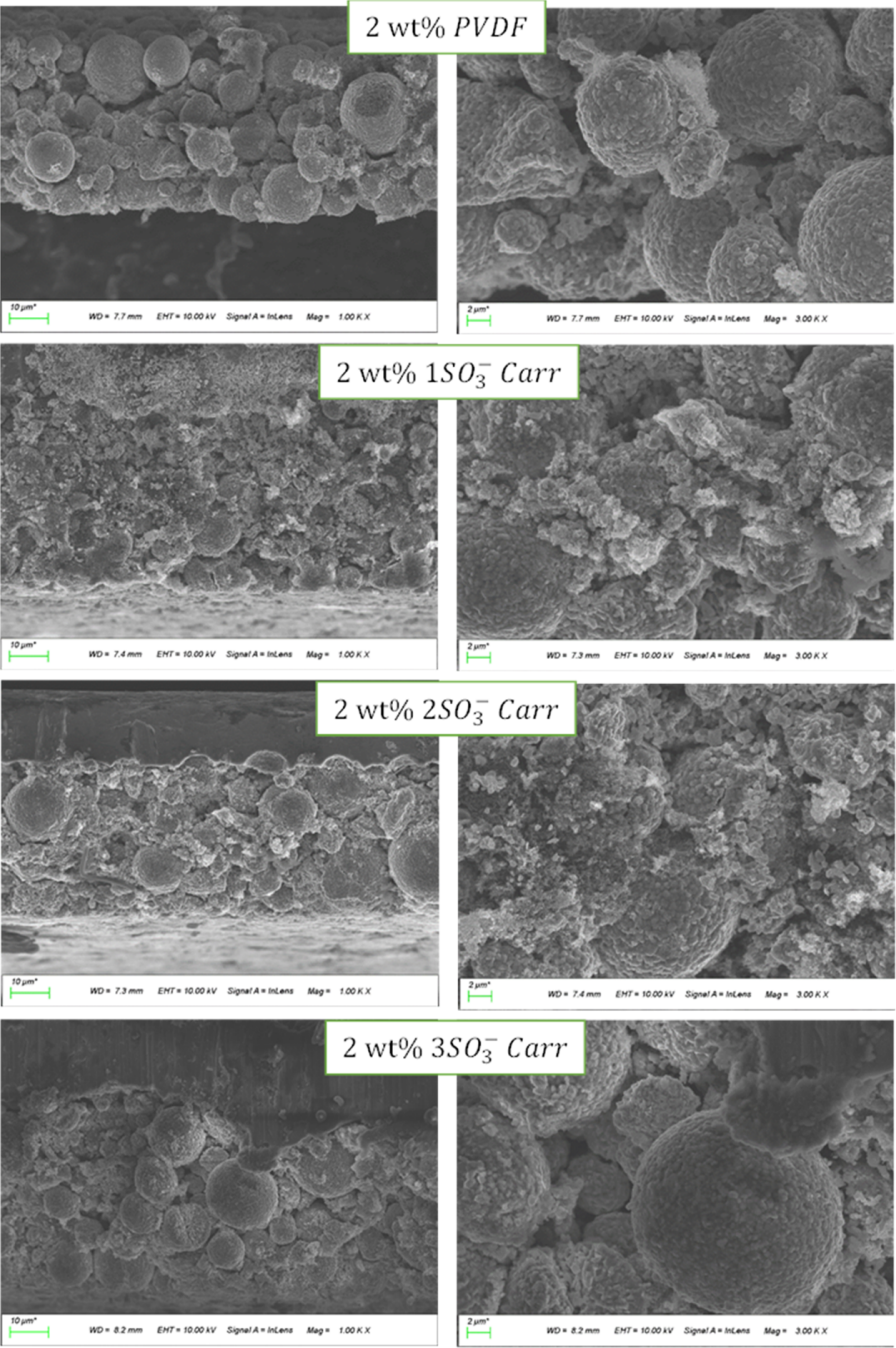
FESEM cross-sectional images of aged electrodes using different binders at 1000× (left) and 3000× (right) magnifications.

Finally, to further explain the effect of different binders on the battery performance, the coin cells were opened to visualize the electrode conditions after cycling. Figure 6 shows the morphology of the electrodes using different binders at 1000× and 3000× magnifications. For the PVDF electrodes, the NMC811 particles presented a spherical shape and no agglomerates were observed. On the other hand, the 1SO_3_^–^*Carr* and 2SO_3_^–^*Carr* electrodes showed degradation with deposition products and protrusions all along the electrodes. This can lead to an increased resistance inside the cell, leading to the capacity fading observed in the galvanostatic cycling. Unlike the less sulfonated carrageenans, the 3SO_3_^–^*Carr* proffered a smooth particle surface with reduced degradation and similar appearance as seen for the organic PVDF coatings, even though the 3SO_3_^–^*Carr* coating was processed in water. Also, cross-sectional FESEM images were acquired of the pristine electrodes, i.e., after the calendaring with no cycling (Figure S6), where no major differences were noted compared with the aged electrodes. The 1SO_3_^–^ and 2SO_3_^–^*Carr* cathodes depicted a damaged morphology, while the 3SO_3_^–^*Carr* looked more like the organic-based PVDF electrode.

## Conclusions

In this work, carrageenan biopolymers were applied as water-soluble binders for NMC811 cathodes, possessing 1, 2, or 3SO_3_^–^ functionalities per unit of the biopolymer. In addition to analyzing the effect of the number of sulfonate groups, we also explored different formulations of binder contents: 5, 2, and 1 wt %. Decreasing the amount of binder gives the advantage of increasing the active material and therefore the capacity of the electrode. With 5 wt % of binder, the slurries with 1SO_3_^–^ and 2SO_3_^–^ binders could not be used to form coatings; the slurries were too dense. Consequently, the 2 wt % binder formulation ensured optimal coating of the electrodes. Finally, in the 1 wt % case, the binder content was too low to generate electrostatic repulsion between particles, and agglomerates were observed.

Therefore, the assessment of the type of carrageenan and the effect of the number of sulfonate groups in its structure was performed with the 2 wt % binder formulations. Of particular notice was the reduction in the charge-transfer resistance over cycling, which was attributed to the formation of more reaction sites. Among these biopolymers, the lambda carrageenan binder, having 3SO_3_^–^ groups, demonstrated significantly improved dispersion properties, adhesion strength, and preservation of the NMC811 active material when exposed to water. The higher content of sulfonate groups in the structure boosted the diffusion kinetics, enabling the 3SO_3_^–^*Carr*-based electrode to deliver higher and more stable discharge capacities. It was able to deliver 133.1 mAh g^–1^ at 3C and 105.0 mAh g^–1^ at 5C, which was similar to the organic-based PVDF electrode (136.1 and 108.7 mAh g^–1^, respectively), while providing a more sustainable route to cathode electrode preparations using a water-soluble, environmentally friendly, and natural polymer. Moreover, the 3SO_3_^–^ carrageenan binder enabled higher energy densities by the reduction of binder amount to 2 wt %, consequently increasing the amount of NMC811 loading, in contrast to PVDF, where 2 wt % of binder decreased the performance capability compared to the 5 wt % binder content.

## Supporting Information Available

Slurries with 5 wt % of binder prepared with 1SO_3_^–^*Carr*, 2SO_3_^–^, and 3SO_3_^–^*Carr*; galvanostatic cycling of 5 wt % of binder electrodes; slurry prepared with 1 wt % of binder formulation; fitting values of the EIS spectra; and FESEM cross sections of pristine electrodes using different binders (PDF)
