# Supplementary material for: Carrageenans as Sustainable Water-Processable Binders for High-Voltage NMC811 Cathodes
Source: ACS Appl Energy Mater. 2023 Aug 14;6(16):8616–25. doi: 10.1021/acsaem.3c01662 (PMC10466266; doi:10.1021/acsaem.3c01662)
Supplement: Supplementary file 1 — ae3c01662_si_001.pdf [file ae3c01662_si_001.pdf]

Supporting information

Carrageenans as Sustainable water-processable Binders for High-Voltage NMC811 cathodes

Ana Clara Rolandi [a,b,c], *Cristina Pozo-Gonzalo*[a], *Iratxe de Meatza*[b], *Nerea Casado*[c,d],

Maria Forsyth[a,c,d*] *and David Mecerreyes*[c,d,*]

[a] Institute for Frontier Materials, Deakin University, Melbourne, Australia 3125

[b] CIDETEC Basque Research and Technology Alliance (BRTA), Paseo Miramon 196, 20014 Donostia-San Sebastian, Spain

[c] POLYMAT, University of the Basque Country UPV/EHU, Avenida Tolosa 72, Donostia-San Sebastián 20018, Spain.

[d]IKERBASQUE, Basque Foundation for Science, Bilbao, 48011 Spain

*To whom correspondence should be addressed.

Email: david.mecerreyes@ehu.es

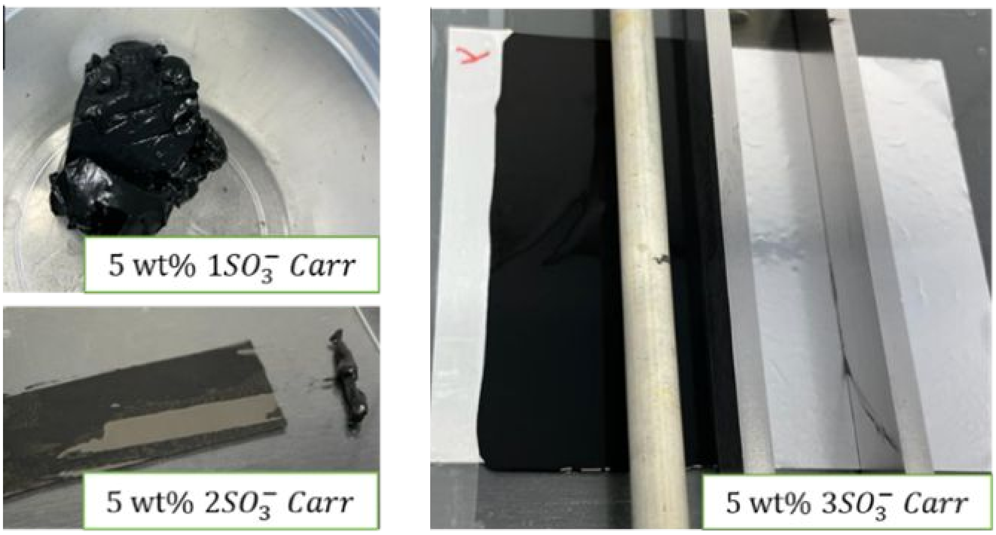

Figure S1. Slurries with 5 wt% of binder prepared with $1SO_3^- \ Carr$, $2SO_3^- \ Carr$ and $3SO_3^- \ Carr$.

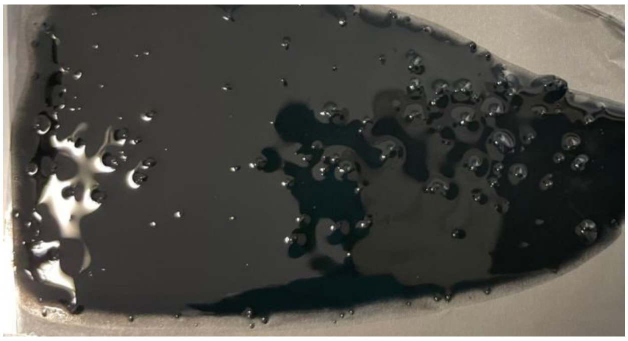

Figure S2. Slurries prepared with the 1 wt% formulation

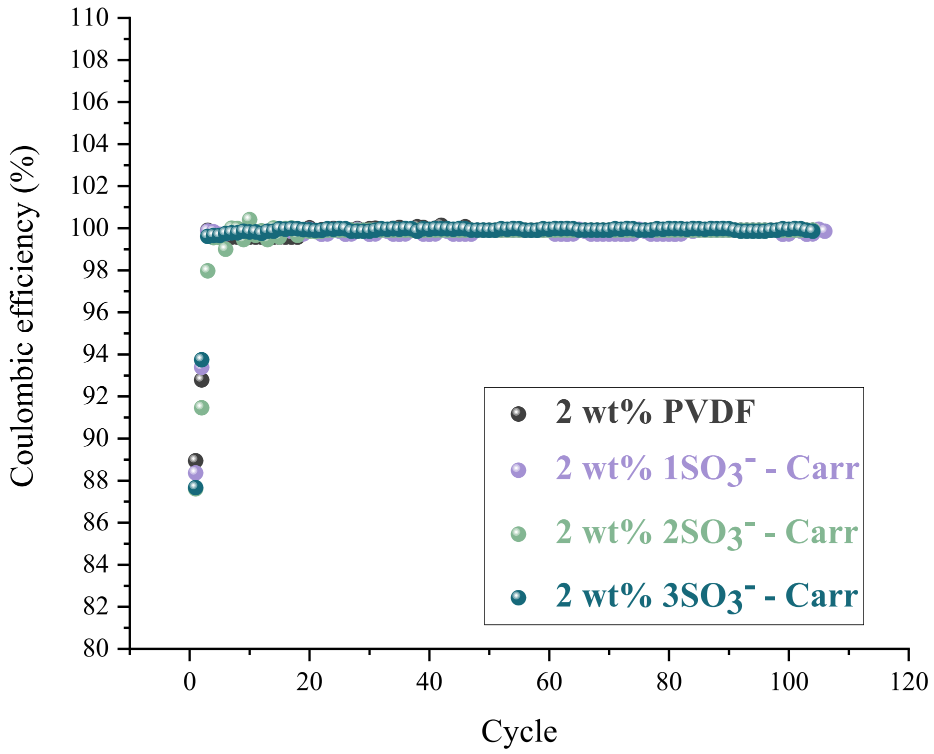

Figure S3. Coulombic efficiency of the galvanostatic cycling of full coin cells prepared from the 2 wt% binder cathode formulations (loading 2.1 mAh cm^{-2}) using different binders.

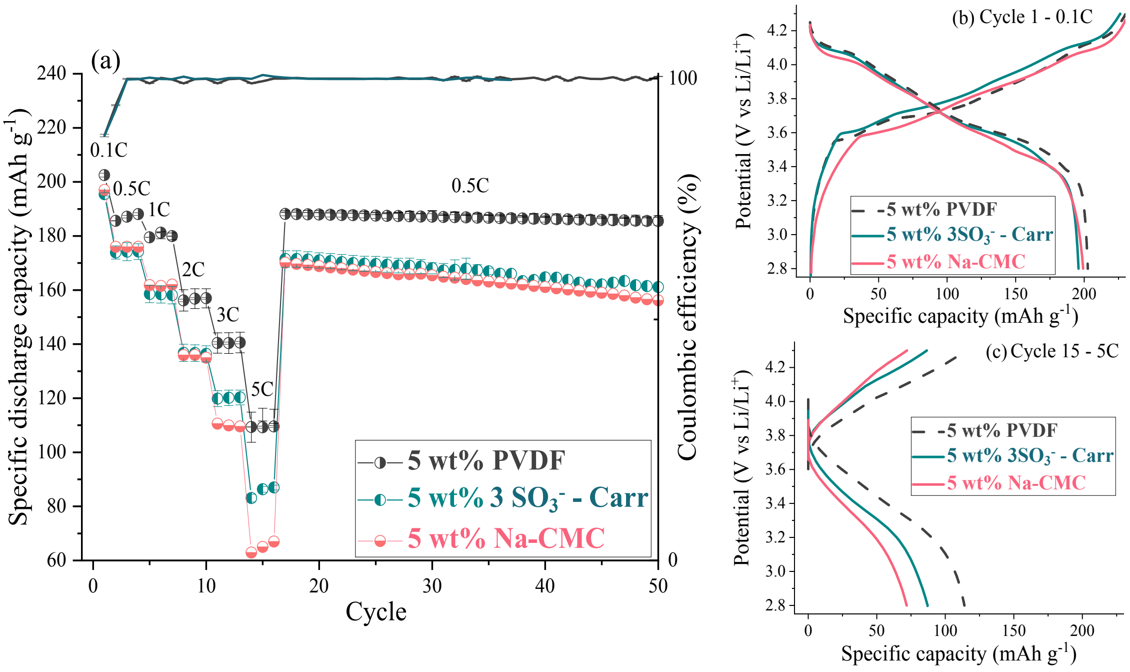

Figure S4. (a) Galvanostatic cycling of coin cells (loading 2.1 mAh cm^{-2}) prepared with the 5 wt% binder formulations, using PVDF and $3SO_3^-\ Carr$ as binders. Voltage profiles at (b) 0.1C and (C) 5C. Potential range: 2.8 - 4.3 V at 25 ˚C.

Table S1. Fitting values of the EIS spectra of NMC811 coin cells using different binders

		R1 (Ω)	R3 (Ω)	σ (Ω $s^{-0.5}$)	D_{Li+}(cm^2s^{-1})
2 wt% PVDF	After forming	1.4 ± 0.1	79.7 ± 4.2	1.6	3.0E-13
	After cycling	3.1 ± 0.9	190.5 ± 10.1	8.1	1.2E-14
2 wt% $1SO_3^-$ $Carr$	After forming	2.1 ± 0.3	435.8 ± 5.6	7.4	1.4E-14
	After cycling	1.4 ± 0.2	171.2 ± 20.2	7.3	1.4E-14
2 wt% $2SO_3^-$ $Carr$	After forming	1.7 ± 0.4	205.9 ± 13.5	9.7	8.0E-15
	After cycling	2.0 ± 0.3	169.5 ± 15.7	6.7	1.7E-14
2 wt% $3SO_3^-$ $Carr$	After forming	1.9 ± 0.1	190.3 ± 8.1	3.0	8.5E-14
	After cycling	1.6 ± 0.1	93.1 ± 4.6	1.8	2.3E-13

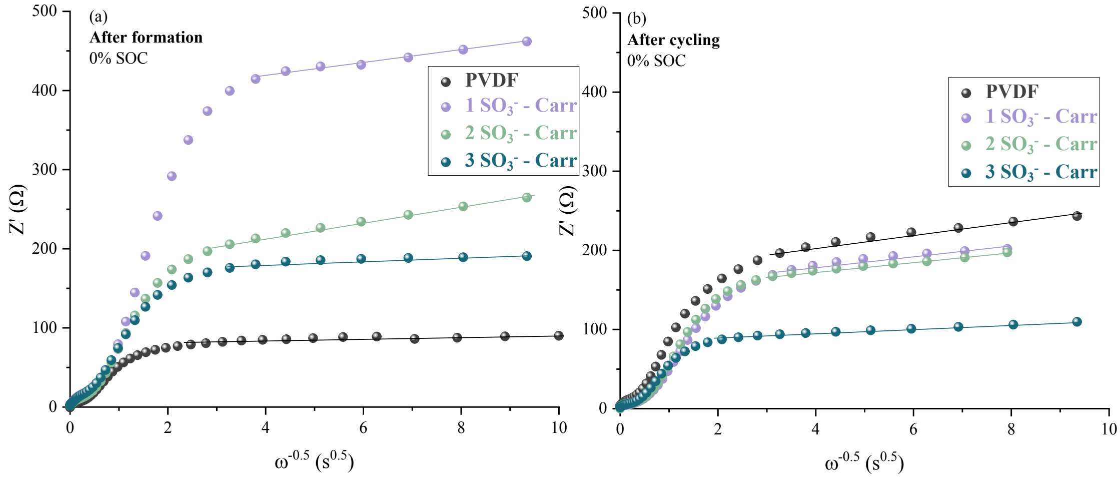

Figure S5. Z' vs $\omega^{-0.5}$ from the EIS measurements on full coin cells (NMC811|Graphite) (a) after the formation step and (b) at the end of cycling (C-rate cycling and 90 cycles at 0.5C) coin cells.

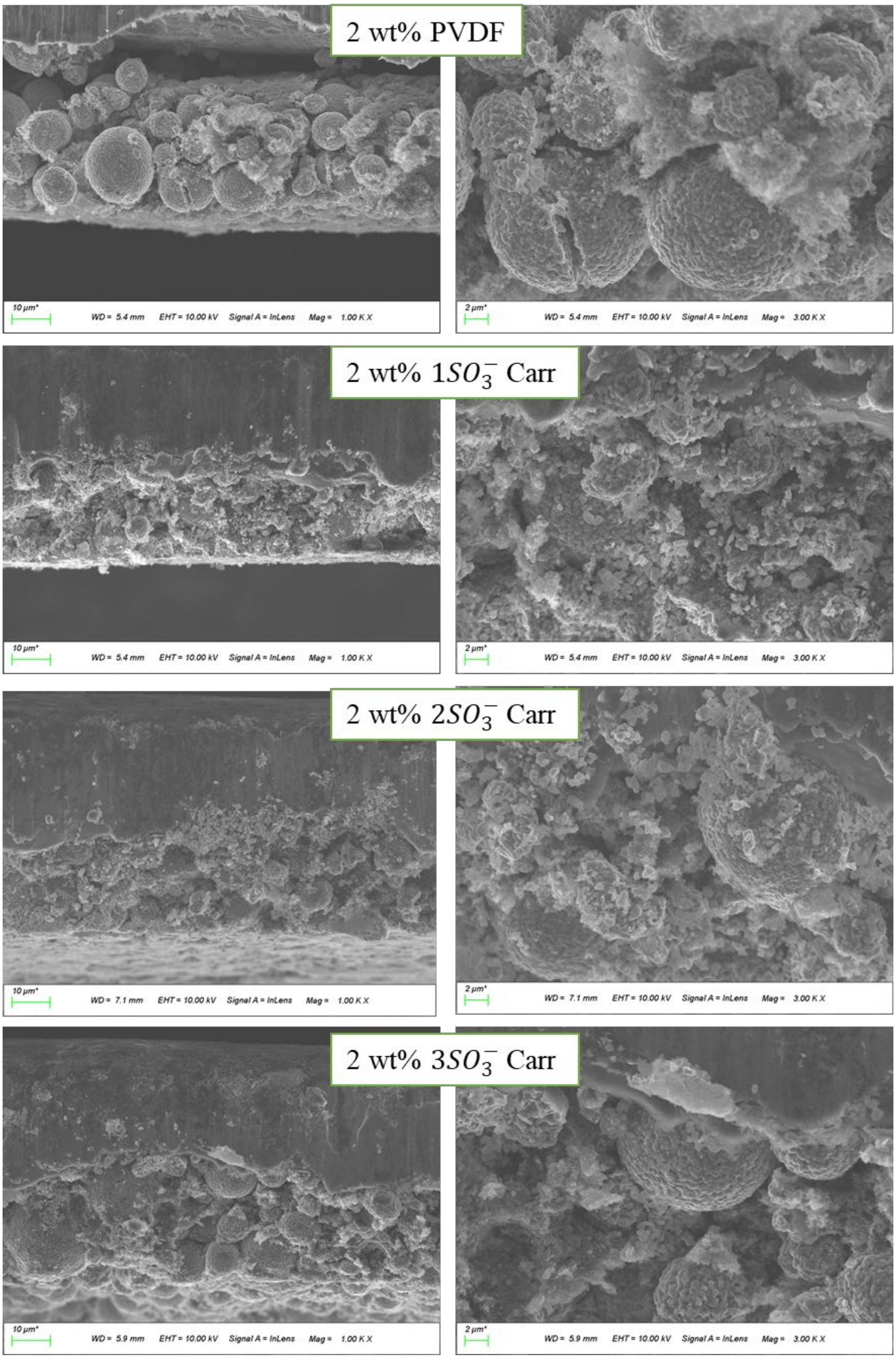

Figure S6. FESEM cross sections images of pristine electrodes using different binders at 1000X (left) and 3000X (right) magnification
